# Supplementary material for: Melon: metagenomic long-read-based taxonomic identification and quantification using marker genes
Source: Genome Biol. 2024 Aug 19;25:226. doi: 10.1186/s13059-024-03363-y (PMC11331721; doi:10.1186/s13059-024-03363-y)
Supplement: Supplementary file 2 — Additional file 2. Supplementary Tables S1–3, Supplementary Figures S1–4, Supplementary Notes S1–3. [file 13059_2024_3363_MOESM2_ESM.pdf]

Melon: metagenomic long-read-based taxonomic identification and quantification using marker genes

Supplementary information

List of Tables

S1 Computational time and peak memory usage for wastewater samples 2
S2 Performance of PHMMs at different scales of threshold scores . . . 3
S3 Statistics of mock and wastewater samples 4
S4 Error rates of models trained on mock samples 5

List of Figures

S1 Comparison between theoretical and expected taxonomic composition of mock communities D6300 and D6331 6
S2 Performance of Melon at different length cutoffs of flanking regions 7
S3 Estimated species-level ARG abundances of mock sample S3 8
S4 Estimated genome copies of PacBio samples 9
S5 ANI between marker-gene-containing sequences 10
S6 Comparison of taxonomic assignment strategies 11
S7 ARG abundances estimated using short and long reads 13

Supplementary Tables

Table S1: Computational time and peak memory usage for wastewater samples

		no pre-filter	PlusPF-8	PlusPF-16	PlusPF[b]
influent 7.816 Gb	genome copy	1,809	1,801	1,800	1,797
	species richness	2,101	2,098	2,099	2,097
	number of filtered reads	-	8,312	10,212	14,988
	mean genome size (Mb)	4.322	4.304	4.300	4.292
	ARG abundance (copies per cell)[a]	0.551	0.553	0.553	0.554
	real time (sec)[c]	2,056 967	2,271 1,027	2,279 1,151	- 1,272
	peak resident set size (GB)[c]	10.629 17.860	10.665 18.143	17.294 17.986	- 77.694
effluent 5.158 Gb	genome copy	1,348	1,336	1,331	1,315
	species richness	1,704	1,697	1,700	1,696
	number of filtered reads	-	16,602	29,300	54,774
	mean genome size (Mb)	3.826	3.789	3.757	3.715
	ARG abundance (copies per cell)[a]	0.507	0.511	0.512	0.519
	real time (sec)[c]	1,341 671	1,496 885	1,495 867	- 929
	peak resident set size (GB)[c]	10.893 13.649	10.902 13.227	17.067 17.454	- 77.325

[a] Excluding multidrug ARGs.
[b] Not tested with MacBook Pro due to insufficient memory.
[c] Excluding ARG abundance estimation, measured with GNU 'time'. Red: MacBook Pro 2021, with Apple M1 Max, 64 GB memory, and macOS Sonoma 14.0. Black: Lab-scale workstation, with 2 × Intel Xeon Silver 4210R CPU 2.40GHz (10 cores, 20 threads), 512 GB memory, and Ubuntu 20.04 LTS.

Table S2: Performance of PHMMs at different scales of threshold scores

	scale	precision	recall	$F_{0.5}$-score	F_1-score	RPGF[a]
bacteria	0.50	0.969	0.931	0.954	0.942	42
	**0.75**	**0.983**	**0.961**	**0.977**	**0.969**	**45**
	1.00	0.984	0.958	**0.977**	0.968	**45**
archaea	0.50	0.920	0.677	0.800	0.734	21
	**0.75**	**0.936**	**0.703**	**0.809**	**0.749**	**23**
	1.00	0.906	0.639	0.750	0.688	19

[a] Number of RPGFs with PHMM's $F_{0.5}$-scores greater than 0.99.

Table S3: Statistics of mock and wastewater samples

					length				quality score	
platform	mock	sample	read	Gb[a]	longest	shortest	mean	median	mean	median
ONT	D6300	S1	2,503,848	16.619	185,010	1,000	6,637	3,947	10.899	11.037
		S2	2,119,258	9.514	51,733	1,000	4,489	3,673	12.085	12.044
		S3	336,330	3.486	211,938	1,000	10,365	3,879	13.187	13.320
	D6331	G1	5,650,723	27.891	50,325	1,000	4,935	4,569	13.742	14.243
		G2	1,570,234	6.458	51,516	1,000	4,112	3,581	16.987	17.321
		G3	713,445	3.587	38,817	1,000	5,028	4,171	19.045	19.365
	-	influent[b]	1,091,772	7.816	374,946	1,000	7,158	6,295	18.320	18.677
		effluent[b]	1,118,502	5.158	91,897	1,000	4,611	3,443	18.211	18.539
PacBio	MSA-1003	-	2,418,889	20.544	21,547	1,001	8,493	8,310	38.768	34.784
	D6331	-	1,978,476	17.993	39,601	1,003	9,094	8,078	45.521	39.623

[a] Gigabase pair (10^9 base pairs).
[b] Two spike-ins species *Allobacillus halotolerans* and *Imtechella halotolerans* were removed before calculation.

Table S4: Error rates of models trained on mock samples

mock	sample	mismatch	insertion	deletion	**total**
D6300	S1	0.040	0.015	0.025	**0.080**
	S2	0.026	0.024	0.032	**0.082**
	S3	0.021	0.014	0.017	**0.052**
D6331	G1	0.017	0.011	0.019	**0.046**
	G2	0.010	0.006	0.013	**0.029**
	G3	0.009	0.006	0.007	**0.022**

Supplementary Figures

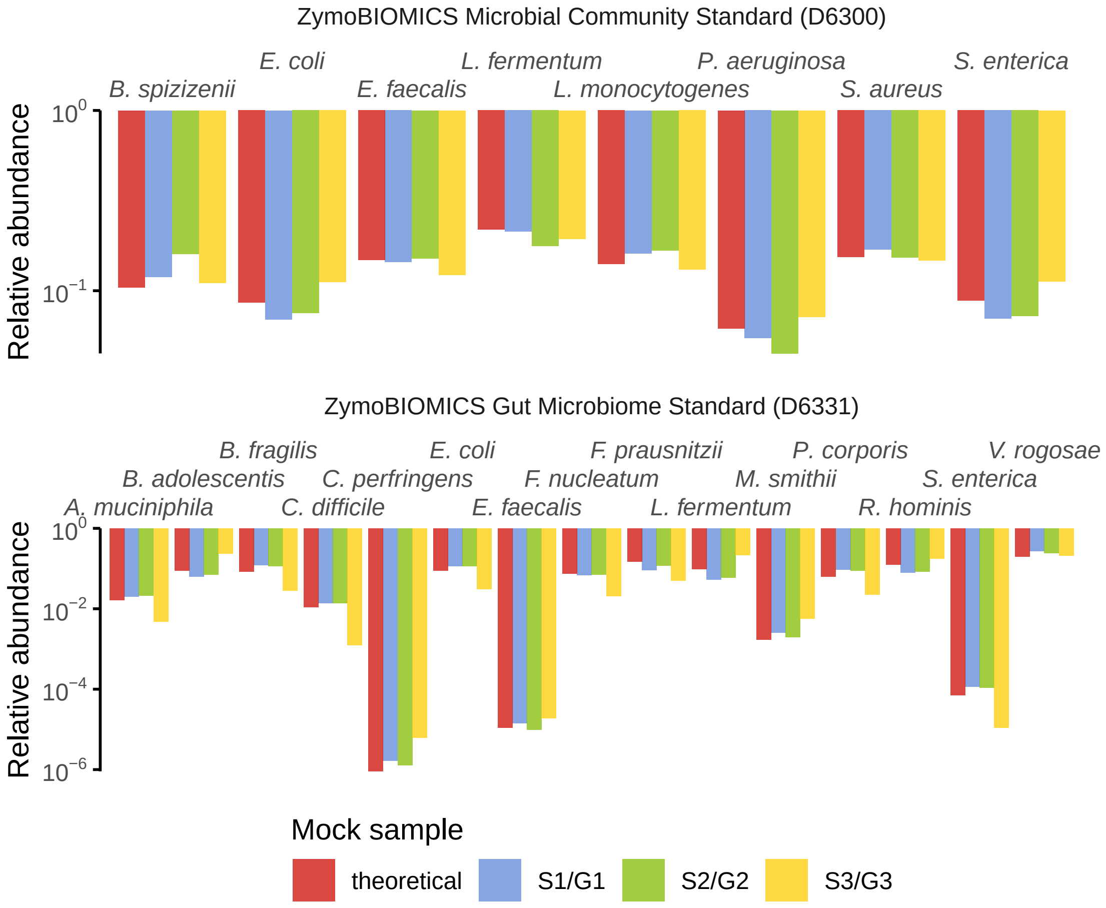

Fig. S1: Comparison between theoretical and expected taxonomic composition of mock communities D6300 and D6331. Theoretical relative abundances (in terms of genome copies) were provided by ZymoBIOMICS. Expected relative abundances were obtained by mapping reads to their associated reference genomes. Yeasts were excluded in both theoretical and expected abundances. Plasmids were not counted while computing expected relative abundances. *Bacillus subtilis* and *Lactobacillus fermentum* have been renamed to *Bacillus spizizenii* and *Limosilactobacillus fermentum*, respectively.

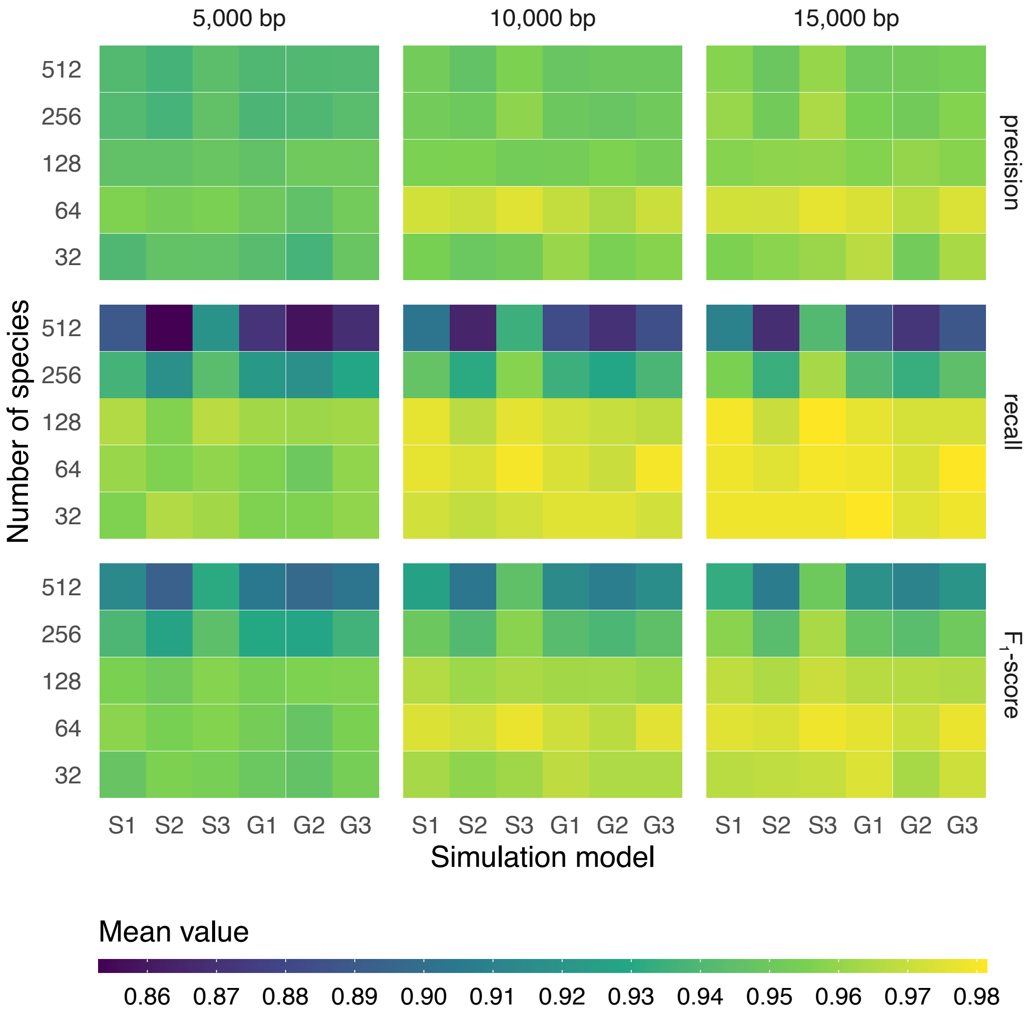

Fig. S2: Performance of Melon at different length cutoffs of flanking regions. Models used for simulation were trained using six mock samples (S1–3 and G1–3). Each combination of simulation models and numbers of species contains ten randomly generated profiles. Colors represent the mean values of these profiles.

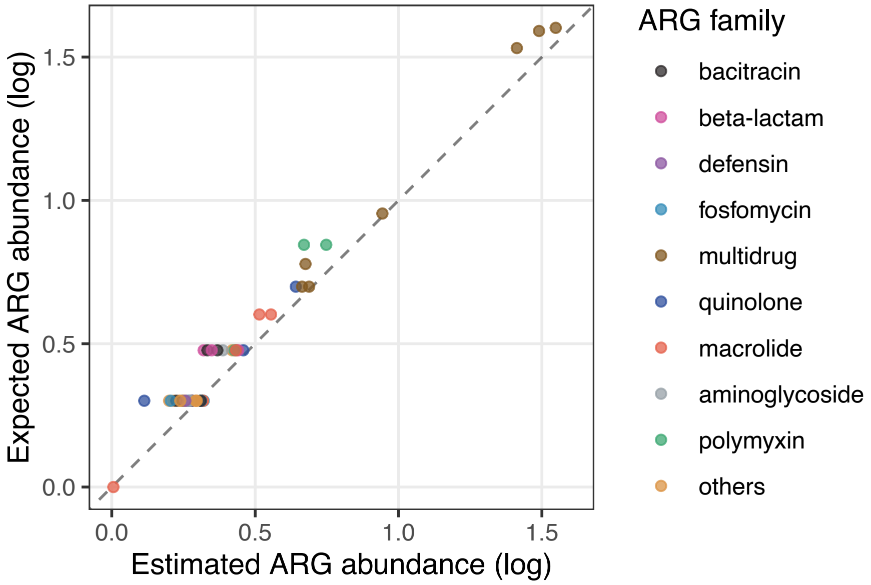

Fig. S3: Estimated species-level ARG abundances of mock sample S3. Estimated species-level ARG abundances (expressed as "copies per cell", assuming one genome copy per cell) were computed by normalising the estimated copies of ARGs by the estimated genome copies provided by Melon. Expected ARG abundances were obtained from the reference genomes given by ZymoBIOMICS. Colors indicate the families of mostly observed ARGs. ARG abundances are shifted by "+1" before $\log_{10}$-transformation to avoid zero entries.

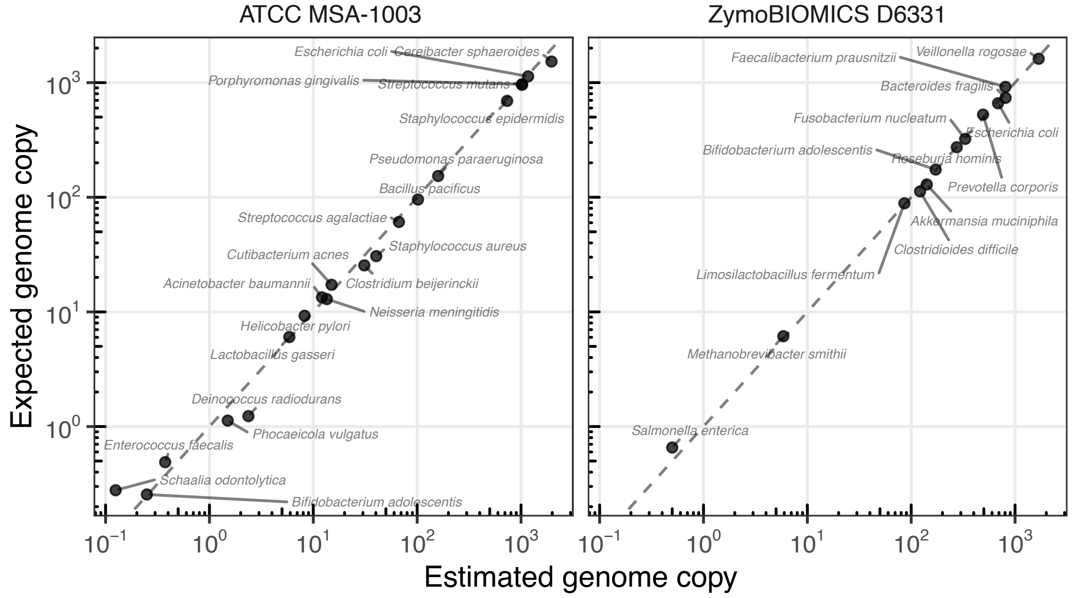

Fig. S4: Estimated genome copies of PacBio samples. Estimated genome copies were returned by Melon. Expected genome copies were obtained by mapping PacBio reads to their respective reference genomes. Reads that mapped to yeasts or remained unmapped were filtered out. Species are labelled with texts. *Pseudomonas aeruginosa* has been renamed to *Pseudomonas paraeruginosa* and *Lactobacillus fermentum* to *Limosilactobacillus fermentum.*

Supplementary Notes

Note S1: Average nucleotide identity between marker-gene containing sequences

We extracted sequences of at most 10,000 bp that covered the selected marker genes (eight each for bacteria and archaea) from RefSeq assemblies using `DIAMOND v2.1.8` (‘`blastx`’) and `SeqKit v2.5.1` (‘`grep`’). The pairwise average nucleotide identity (ANI) between all marker-gene-containing sequences was computed using `skani v0.2.1` (‘`dist`’). We recorded the identity of a pairwise comparison if the aligned fraction was greater than 75% for both the query and the reference.

As shown in Fig. S5, the median ANI ranged from 99.0% (bacterial s16) to 99.8% (bacterial l2) between strains and from 93.8% (archaeal l2) to 96.9% (archaeal l10e) between species. Given that RPGs are in general more conserved compared to other nonessential genes, it was expected that some between-species ANIs exceeded the conventional ANI cutoff for species identification, i.e., 95%. However, for most marker genes, there was still a gap between the within-species and within-genus ANIs. This large difference in species/genus-level ANIs provided evidence that these marker-gene-containing sequences were sufficient for delivering species-level taxonomic resolution.

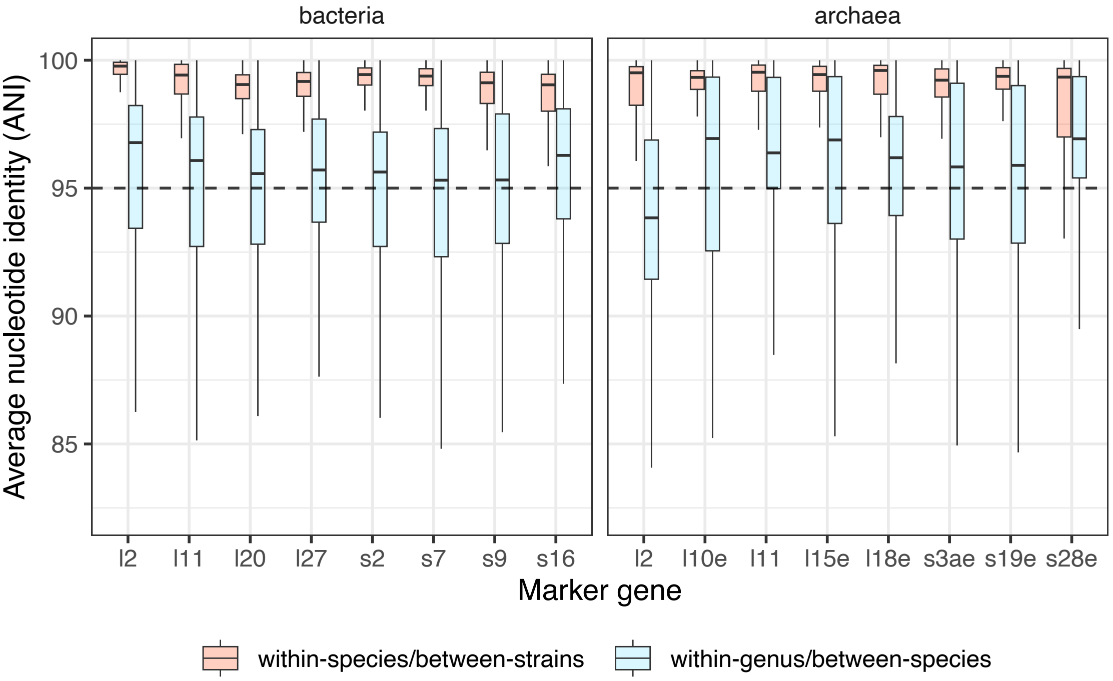

Fig. S5: ANI between marker-gene-containing sequences. Colors indicate within-species/within-genus identity. Dashed lines represent the 95% ANI cutoff for species identification. Archaeal ribosomal protein genes s2, s7, and s9 were not included.

Note S2: Comparison of taxonomic assignment strategies

We collected the reference genomes of the top 24 pathogens from NCBI (https://www.ncbi.nlm.nih.gov/pathogens/organisms/) and assembled one genome from a wild-type *E. coli* isolated from a wastewater treatment plant. All these genomes were present in the RefSeq database, except for the genome of the wild-type *E. coli*. We simulated two samples with the 25 genomes (even taxonomic abundance) using `NanoSim`: high-quality (HQ, profile G3) and low-quality (LQ, profile S3), each with 500,000 reads. We specifically used the marker-gene-containing reads to see whether various taxonomic classification strategies could correctly identify their taxonomy. For evaluation, we focused on the percentage of misclassified reads. The types of misclassification include (1) true positive (the misclassified species is within the 25 species), (2) false positive (the misclassified species is not among the 25 species), and (3) unclassified (the read is not utilized by the classifier or the classification does not have a species-level taxonomy).

To make a fair comparison, we exclusively used RefSeq complete genomes (collected on May 10, 2024) and rebuilt the database for `Kraken2`, `Centrifuger`, `MetaMaps`, and `minimap2` (the database of `minimap2` is shared by `minimap2+BH`, `minimap2+EM`, and `MEGAN-LR`).

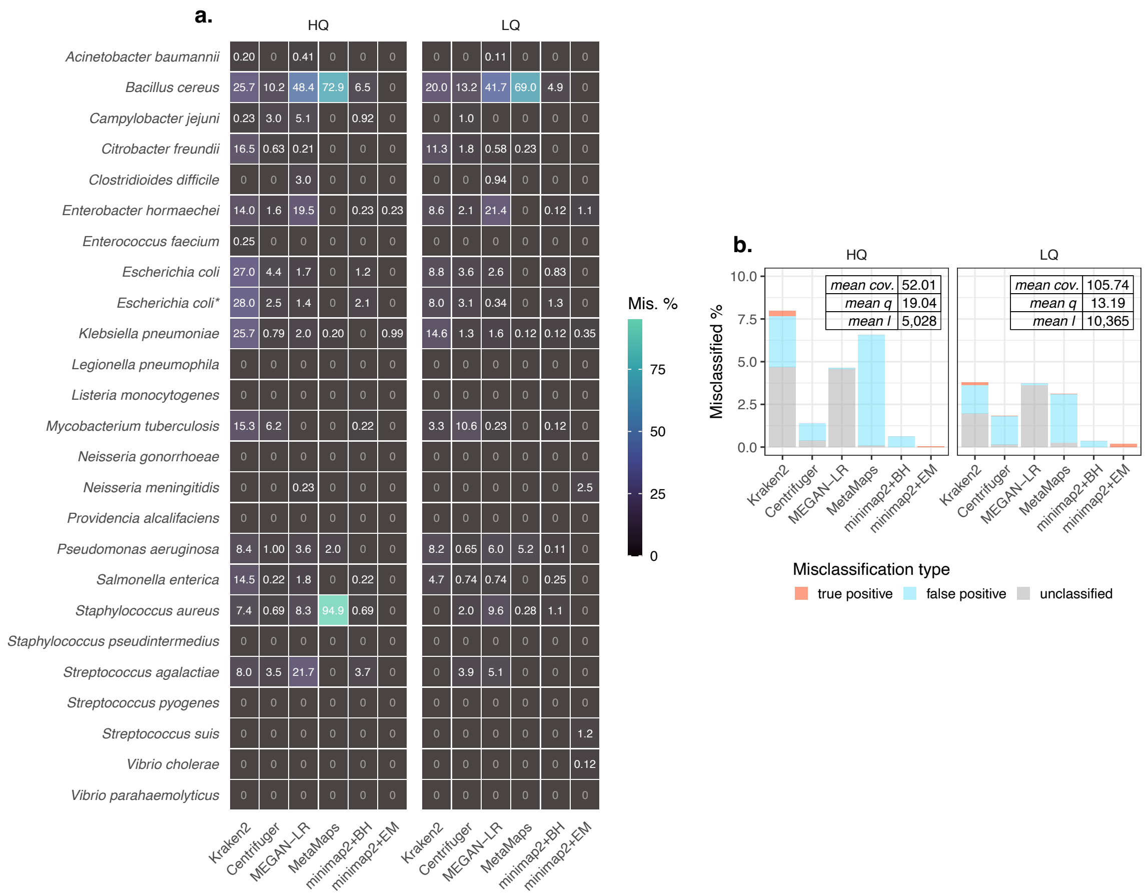

Fig. S6: Comparison of taxonomic assignment strategies. **a.** Species-level misclassification. Color indicates the percentage of misclassified reads (Mis. %). Wild-type *E. coli* is labelled with an asterisk. **b.** Overall misclassification. Color represents types of misclassification. Characteristics of simulation profiles are shown in tables.

Regarding the taxonomic classification strategies, `minimap2` refers to the native base-level alignment of Nanopore reads (preset `map-ont`). `minimap2+BH` retains a single best hit for

each read based on the alignment score (AS) and adopts its taxonomy. `minimap2+EM` is the taxonomic assignment strategy of `Melon`, where taxonomic labels are reassigned with EM. `MEGAN-LR` aggregates the alignments of `minimap2` using a lowest common ancestor (LCA) algorithm called interval-union LCA (note that `MEGAN-LR` can run with either a protein or a nucleotide database and by default uses `DIAMOND` with NCBI nr and `minimap2` with NCBI nt). `MetaMaps` employs minimizer-based approximate mapping and EM for post-correction. `Kraken2` and `Centrifuger` are both k-mer-based (or similar) alignment-free methods.

In Fig. S6, we see that `minimap2+BH` and `minimap2+EM` clearly outperformed all the other methods. `MEGAN-LR` also employs `minimap2` but its LCA algorithm made many reads unclassified at species level. `Kraken2` showed the worst performance and its accuracy varied strongly across species. Unlike `Kraken2`, `Centrifuger` performed reasonably well despite also being alignment-free. `MetaMaps` could not correctly classify certain species, e.g., *Bacillus cereus* and *Staphylococcus aureus*, resulting in much worse overall performance compared to `minimap2+EM` (note that the implementation of EM may not be exactly identical). Interestingly, we observed that a higher quality of reads did not always lead to better classification for all classifiers except for `Centrifuger`. This suggests that these classifiers are less sensitive to quality score q but more sensitive to read length l. We also see that the additional EM step (`minimap2+EM`) greatly reduced the number of false-positive misclassifications but might accidentally skew the relative abundance estimate by introducing true-positive misclassifications due to the presence of highly similar species (e.g., *Neisseria meningitidis* being classified as *Neisseria gonorrhoeae*, ANI 94.94%). This experiment indicated the advantages of using base-level alignment plus post-error correction for accurate taxonomic labelling of long reads.

Note S3: Validation of ARG quantification

We downloaded and reanalysed the 109 Singaporean human faecal samples (paired Nanopore and Illumina data) from PRJEB49168 using both the strategy we presented and `ARGs-OAP v3.2.4`. `ARGs-OAP` is a short-read-based ARG quantification pipeline. Since `ARGs-OAP` does not provide species-level taxonomic information, we compared the total ARG abundance (expressed as ARG copies per genome copy) of the samples. As shown in Fig. S7, the estimated ARG abundances were highly correlated (Spearman's correlation $\rho = 0.941$, permutation test, two-sided, $p < 0.001$).

Despite being highly correlated, the ARG abundances estimated by long reads were systematically higher due to: (1) the database we used in this manuscript was the *full* version of the SARG database, which includes additional genes such as transcriptional regulators (e.g., activators and repressors) compared to `ARGs-OAP`'s *short* version; (2) `ARGs-OAP` considers two- or three-component systems (e.g., *arcA-arcB-tolC*, the primary efflux pump of *E. coli*) as single units. These genes are weighted by a factor of either 1/2 or 1/3, leading to a significant reduction in ARG abundance estimation; and (3) the cutoffs we employed for long reads (identity 75% and subject cover 75%) may not be directly comparable to those used by `ARGs-OAP` (identity 80% and query cover 85%).

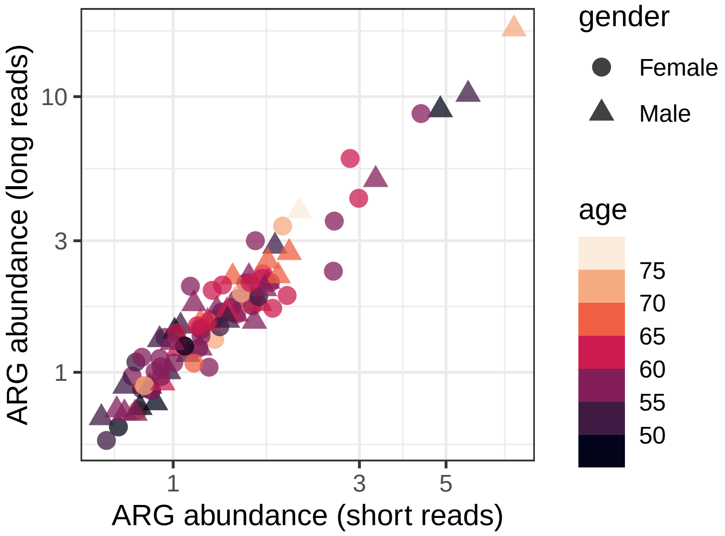

Fig. S7: ARG abundances estimated using short and long reads. Colors and shapes indicate subjects' age and gender, respectively.
